# Case Report: A case of drug-induced pancreatitis caused by paroxetine with a literature review

**DOI:** 10.3389/fmed.2025.1688065

**Published:** 2026-01-05

**Authors:** Yangpeng Wu, DongFeng Lv, Lihua Liu, YaDong Liu

**Affiliations:** Department of Emergency, Xi’an No. 3 Hospital, Xi’an, Shaanxi, China

**Keywords:** paroxetine, acute pancreatitis, drug-induced pancreatitis, antidepressants, case report

## Abstract

**Background:**

Acute pancreatitis (AP) incidence is ~76.2 per 100,000. Drug-induced pancreatitis (DIP) accounts for <2%, yet >500 drugs are implicated. Evidence linking the SSRI paroxetine to AP remains sparse.

**Case presentation:**

A 28-year-old woman with severe depression self-initiated paroxetine 20 mg once daily; within 24 h she developed persistent epigastric pain and bloating. After brief relief with intravenous fluids she re-administered the same dose, and symptoms recurred within 24 h. Labs showed elevated pancreatic enzymes and CRP. CT revealed mild pancreatitis. No alcohol, hyperlipidemia or other classic risk factors were identified. Paroxetine was immediately discontinued; supportive care led to symptom and enzyme normalization within 72 h. She was discharged on trazodone and remains recurrence-free.

**Discussion:**

Population studies have not established a significant SSRI–AP association, probably because of the extreme rarity of such events (<0.01%). The positive rechallenge observed here provides robust individual-level evidence of causality, supported by formal probability scales and international criteria. SSRIs may precipitate AP through serotonin-mediated *β*-cell dysfunction, oxidative stress, or idiosyncratic hypersensitivity. Early recognition is critical because DIP is usually reversible after drug withdrawal.

**Conclusion:**

This case suggests that paroxetine may induce AP even at standard doses. Clinicians should consider DIP in patients lacking traditional risk factors, especially after self-rechallenge. Early recognition, immediate drug cessation and supportive therapy ensure excellent recovery.

## Introduction

Acute pancreatitis (AP) is a common and potentially severe abdominal disorder with a rising global incidence. The most frequent etiologies include biliary tract disease, alcohol abuse and hyperlipidemia (1). While DIP accounts for less than 2% of AP cases, more than 500 medications—including various antidepressants—have been identified as potential triggers (2–4). Antidepressants, particularly selective serotonin reuptake inhibitors (SSRIs), are widely prescribed for psychiatric disorders and are generally considered safe. However, serious adverse reactions such as DIP, though rare, can occur and may be underrecognized due to nonspecific clinical presentations (5, 6). DIP is often overlooked, with the highest risk typically occurring within the first 2 weeks after drug initiation (3, 7).

The diagnosis of DIP is challenging, often requiring the exclusion of common etiologies and establishing a temporal relationship between drug exposure and symptom onset. Paroxetine, a SSRI, is widely used for the treatment of depression and anxiety disorders. The clinical course, risk factors and pathophysiological mechanisms underlying SSRI-induced pancreatitis are not fully understood (6, 8).

This report adheres to the CARE guidelines, with a completed checklist provided as Supplementary material. We report a case of paroxetine-induced AP in a young woman, in which other causes were systematically excluded. Notably, the recurrence of symptoms upon patient-initiated rechallenge with paroxetine provides robust individual-level evidence supporting causality. This case highlights the importance of considering DIP in patients presenting with AP without traditional risk factors, especially when there is a clear temporal association with drug initiation. Withdrawal of the drug and supportive care resulted in full recovery, with no recurrence during follow-up.

## Case presentation

A 28-year-old Chinese woman presented to the emergency department of Xi’an No. 3 Hospital on March 8, 2024, with escalating epigastric pain that had persisted for 1 week. She had a history of major depressive disorder diagnosed 1 year prior, manifesting as persistent low mood, anhedonia, insomnia and easy fatigability, for which she intermittently used clonazepam at night as needed. Seven days before admission (March 1, 2024), she obtained paroxetine 20 mg tablets from a fellow patient and began self-administering one tablet daily without professional consultation. Within 24 h of the first dose, she developed persistent dull upper-abdominal pain and bloating. She sought care at a local clinic, where she received symptomatic treatment and intravenous fluids, which led to only transient improvement. Three days before admission (March 5, 2024), without medical guidance, she resumed the same dose of paroxetine, and within 24 h her pain recurred and intensified. She denied any history of pancreatitis, gallstones, hypertension, diabetes, hyperparathyroidism, hypertriglyceridemia, cardiovascular disease, trauma, or relevant surgical procedures including ERCP. She also reported no alcohol use, smoking, unhealthy diet, high-fat intake, familial hyperlipidemia, or drug/food allergies.

**Figure 1:**
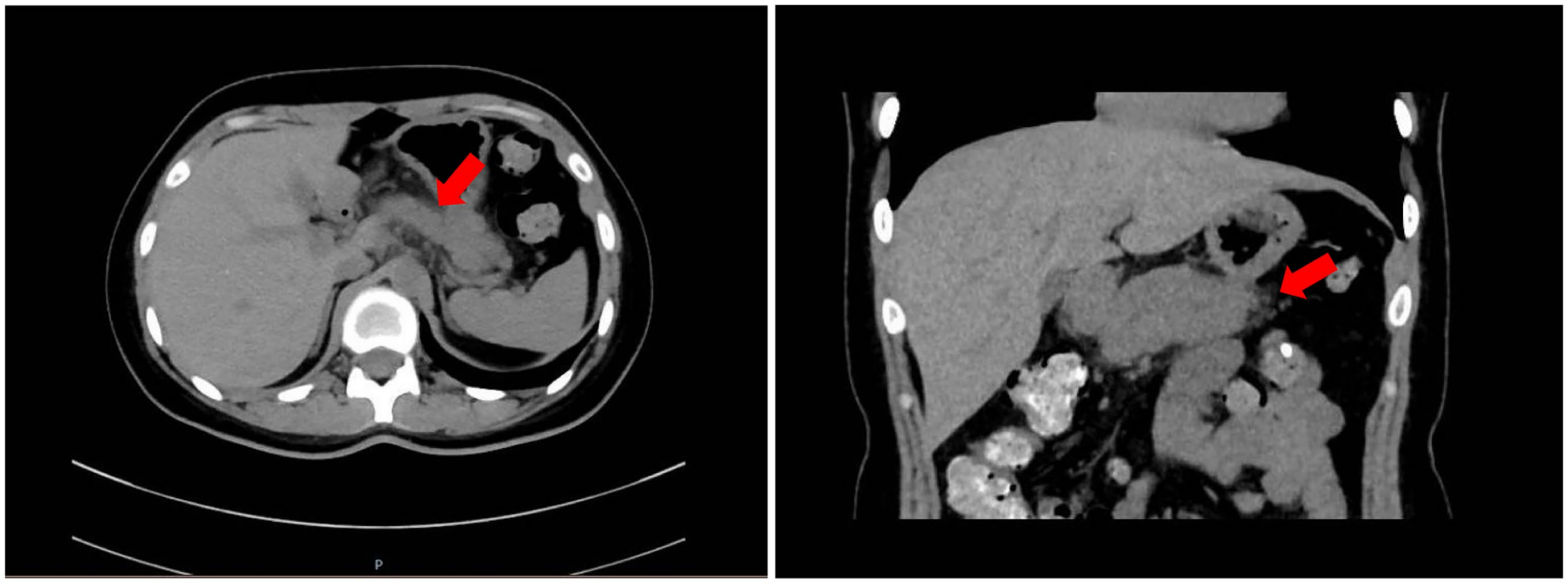
Abdominal CT (7 March 2024) showing ill-defined peripancreatic fat stranding (arrows) and mild enlargement of the pancreatic body (arrowhead); no biliary dilatation or cholelithiasis was present.

On arrival she was alert but distressed (BMI 18.42 kg/m^2^; BP 90/68 mmHg). Examination revealed mild epigastric tenderness without rebound; bowel sounds were diminished. Laboratory values: amylase 308 U/L, lipase 773.6 U/L (>3 × ULN), C-reactive protein (CRP) 22.39 mg/L; lipids, calcium, liver and renal panels normal (detailed in Table 1). Abdominal CT showed mild pancreatic enlargement with peripancreatic fat stranding and no biliary obstruction (Figure 1). She met revised Atlanta criteria for mild AP (BISAP 0).

**Table 1:** Laboratory findings during hospitalization.

Laboratory test	Day 1 (admission)	Day 4 (follow-up)	Normal range
RBC	4.19 × 10^12^/L	3.63 × 10^12^/L	3.8–5.1
WBC	9.39 × 10^9^/L	3.52 × 10^9^/L	3.5–9.5
Hemoglobin	138 g/L	121 g/L	115–150
Platelet count	300 × 10^9^/L	239 × 10^9^/L	125–350
HCT	39.8%	34.6%	35–45
Neutrophil percentage	60.2%	29.7%	40–75
CRP	22.39 mg/L	10.69 mg/L	0–10
Total protein	72.7 g/L	57.7 g/L	65–85
Albumin	47.9 g/L	36.6 g/L	40–55
Calcium	2.33 mmol/L	2.07 mmol/L	2.11–2.52
Magnesium	0.90 mmol/L	0.78 mmol/L	0.75–1.02
Phosphorus	0.87 mmol/L	1.14 mmol/L	0.85–1.51
Blood urea nitrogen	3.39 mmol/L	1.01 mmol/L	2.6–7.5
Serum creatinine	62 μmol/L	55 μmol/L	41–73
Uric acid	178 μmol/L	181 μmol/L	155–357
Glucose	5.88 mmol/L	5.90 mmol/L	3.9–6.10
Total bilirubin	9.6 μmol/L	5.7 μmol/L	≤21.0
Direct bilirubin	1.9 μmol/L	1.0 μmol/L	0.0–12
Amylase	308 U/L	129 U/L	35–135
Lipase	773.6 U/L	200.6 U/L	5.6–51.3
Total cholesterol	3.42 mmol/L	-	3–5.7
Triglycerides	0.62 mmol/L	-	0–1.70
Alanine aminotransferase	13 U/L	12 U/L	7–40

Reference ranges are based on WS/T 405–2012 standards (National Health Commission of the PRC).

**Figure 2:**
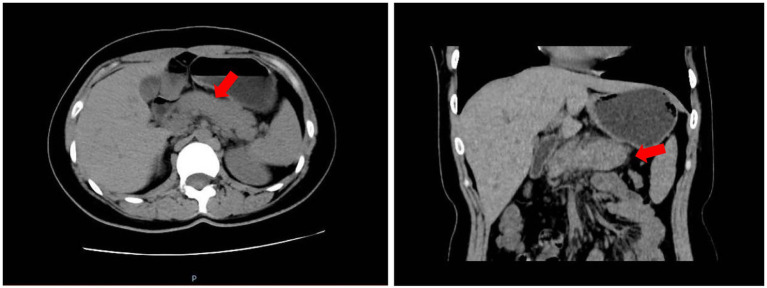
Abdominal CT (13 March 2024, 6 days after drug withdrawal) demonstrating near-complete resolution of peripancreatic exudates and normal pancreatic size.

Upon admission, paroxetine was immediately discontinued. Supportive care—NPO, intravenous fluids, stepwise parenteral-to-enteral nutrition—resulted in rapid clinical improvement. Psychosomatic consultation recommended discontinuing paroxetine due to depressive symptoms; diazepam 10 mg was administered intramuscularly or intravenously as needed for insomnia. Subsequently, trazodone (50–100 mg/night) were prescribed to manage depressive symptoms. By day 3 (March 11), pain had resolved, enzymes had normalized (amylase 129 U/L, lipase 200.6 U/L) and repeat CT showed near-complete resolution of peripancreatic exudates (Figure 2). Laboratory re-evaluation on the same day (Day 4 after admission) showed a slight decrease in neutrophil percentage (29.7%), which was attributed to hemodilution from supportive intravenous fluids; no signs of infection (e.g., fever, elevated CRP) were observed, confirming no additional complications. She was discharged on trazodone under psychiatric follow-up; no recurrence has been documented. The clinical course is summarized in Supplementary Table S1. Written informed consent was obtained from the patient for the publication of this case report and any accompanying images.

## Discussion

SSRIs are widely regarded as safe and effective medications, yet their potential association with AP remains controversial. Population-based studies (9, 10) have not demonstrated a significant link between SSRIs and AP. However, our patient developed definitive pancreatitis following a patient-initiated rechallenge with paroxetine. Several factors may explain this discrepancy. Firstly, although both cited studies included over 60,000 SSRI users, the reported incidence rates were ≤5–6 per 10,000 person-year (10). Such a low frequency (<0.01% of treated subjects) may be obscured in statistical analyses (9). Secondly, these studies pooled multiple SSRIs, varying dosages and concomitant medications, whereas our patient received a single agent (paroxetine 20 mg daily) in the absence of other risk factors such as alcohol use, obesity or hyperlipidemia, potentially identifying a genetically or metabolically susceptible subgroup. Thirdly, reliance on discharge coding in registries may result in under-recognition or misclassification of mild DIP as nonspecific abdominal pain (9, 10). Consequently, while registry data indicate overall safety, they cannot exclude extremely rare but clinically important idiosyncratic reactions such as the present case.

Notably, our patient’s positive rechallenge provides robust individual-level evidence of causality (Naranjo score 7, “probable”; WHO-UMC: “probable/likely”). The detailed scoring is presented in Supplementary Table S2 (Naranjo scale) and Supplementary Table S3 (WHO-UMC criteria). Such reactions are often overlooked in large-scale epidemiological studies, highlighting the need for clinicians to remain vigilant for rare but serious adverse effects, even when population data suggest overall safety. Mechanistically, SSRIs have been implicated in metabolic disturbances, including type II diabetes mellitus and potentially AP (11). Variability in study design, patient populations and pharmacokinetic profiles among different SSRIs may contribute to inconsistent findings. Paroxetine may trigger pancreatic injury through three non-mutually exclusive pathways: Firstly, serotonin-mediated *β*-cell dysfunction. SSRIs block the serotonin reuptake transporter (SERT) in pancreatic islets, increasing extracellular 5-HT and impairing insulin secretion via down-regulation of GLP-1 receptors (8). Secondly, oxidative stress. Elevated 5-HT enhances NADPH oxidase activity, generating reactive oxygen species that damage acinar cell membranes (12). Thirdly, hypersensitivity. Paroxetine is metabolized to highly reactive catechol intermediates that can act as haptens, eliciting a T-cell-mediated inflammatory response in the pancreas (13). Recent studies have suggested that genetic polymorphisms in drug-metabolizing enzymes, particularly cytochrome P450 isoforms such as CYP2D6 and CYP2C19, may significantly influence individual susceptibility to SSRI-induced adverse effects, including pancreatitis (8, 14). Individuals with poor metabolizer phenotypes may experience higher plasma concentrations of paroxetine, potentially increasing the risk of idiosyncratic toxicity (8, 14). In addition, pharmacogenomic screening has been proposed as a potential tool to identify at-risk patients prior to SSRI initiation, although it is not yet routinely implemented in clinical practice. Several biomarkers predictive of DIP, such as elevated baseline serum trypsinogen or pro-inflammatory cytokines (e.g., interleukin-6), are currently under investigation, but their utility in routine risk stratification remains to be validated (7). These findings suggest that differences in drug metabolism and immune response likely contribute to the extreme rarity of DIP, supporting the hypothesis of a multifactorial and idiosyncratic pathogenesis (7, 8, 14). Recent pharmaco-epidemiological data (2022–2024) continue to support an incidence < 0.01%, consistent with an idiosyncratic mechanism (4).

DIP refers to pancreatic inflammation precipitated by medications, their metabolites, or idiosyncratic host responses (15). DIP frequently lacks specific clinical or laboratory features, complicating diagnosis. Most cases are mild or moderate in severity, but severe and fatal outcomes have been reported, with mortality rates as high as 9–15% in the most severe instances (16). Although DIP accounts for a minority of AP cases, over 500 medications, including antineoplastic, anti-infective, immunosuppressive and hormonal agents, have been implicated (17, 18) (Supplementary Table S4). DIP may be underdiagnosed, particularly when misattributed to more common etiologies such as gallstones or alcohol (19).

The latency period for DIP is variable, ranging from hours to over a year, though most cases present within the first week of drug exposure (20). Diagnosis is based on four criteria: thorough exclusion of other common causes (gallstones, alcohol, hypertriglyceridemia, autoimmune disease); clear temporal association between drug initiation and symptom onset; resolution after drug discontinuation; and recurrence upon drug rechallenge. High-risk populations include older adults, women, those with multiple comorbidities and patients on polypharmacy (21). Management centers on immediate withdrawal of the suspected agent and supportive care. Patient education about potential risks is crucial to prevent recurrence.

In this case, a comprehensive evaluation excluded both common and atypical causes of AP. The patient had no personal or family history of hyperlipidemia, alcohol misuse, or high-fat dietary intake. Imaging ruled out gallstones, biliary tract disease and ductal abnormalities. There was no evidence of IgG4-related or other systemic autoimmune disease. Liver function and bilirubin were normal, and there were no signs of infection. Although advanced studies (such as MRCP and IgG4/viral serologies) were not performed, the clinical context favored a non-biliary, non-autoimmune and non-infective etiology. Future cases should consider expanded microbiological and immunological workup as appropriate.

The temporal relationship between paroxetine exposure and symptom onset was further strengthened by the positive patient-initiated rechallenge. While not a physician-supervised rechallenge, the sequence of events, onset after re-exposure, resolution after discontinuation and recurrence upon re-use, which supports a causal relationship according to the Naranjo scale (score: 7, “probable”) and WHO-UMC criteria (“very likely”) (22, 23). We note that this rechallenge occurred outside a controlled setting, reflecting real-world medication behaviors, which provide valuable evidence in spontaneous adverse drug reaction reporting. The reproducibility of symptoms upon repeated paroxetine exposure provides robust evidence for causality, rarely documented in the literature and emphasizes the importance of monitoring and documenting suspected DIP cases.

A review of the literature reveals only a handful of reported cases of paroxetine-induced pancreatitis. Vucicevic et al. described a fatal case of hemorrhagic pancreatitis in a patient receiving multiple psychotropics, including paroxetine (5). Pamukcu et al. reported a young male who developed mild AP within 36 h of high-dose paroxetine, with full recovery after withdrawal (24). Touaoussa et al. documented AP following paroxetine exposure in a patient with hematologic malignancy (25). Few reports have described positive rechallenge, as seen in our patient. This unique aspect provides particularly strong evidence for causality and underscores the need for clinicians to consider antidepressant-induced pancreatitis in cases of unexplained AP. Comparison with previously reported paroxetine-induced AP cases is summarized in Table 2, highlighting the uniqueness of our patient who experienced a positive self-rechallenge at the standard therapeutic dose of 20 mg.

**Table 2:** Paroxetine-induced acute pancreatitis in published data.

No.	Gender/Age	Dose and frequency	Time to onset	Severity of the acute pancreatitis	Comorbidity	Co-drug	Rechallenge	Outcomes	Reference
1	M/44	Not reported in literature	Not reported in literature	Severe	Yes	Yes	Not performed	Death	Vucicevic Z (5)
2	M/23	40 mg, once a day	36 h	Mild	No	No	Not performed	Recovery	Gül Pamukcu (24)
3	M/51	Not reported in literature	15 days	Severe	Yes	Yes	Not performed	Recovery	Aziz Touaoussa (25)
4	F/28	20 mg, once a day	1 day	Mild	No	No	Yes	Recovery	This case

Beyond the individual case, our patient’s self-medication with paroxetine aligns with a global pattern of unsupervised psychotropic use. Population-based surveys across 46 countries report that 11–93% of adults obtain prescription-only medications (including psychotropics) without a physician’s prescription, with common drivers including perceived mild symptom severity, time constraints, unregulated pharmacy access and peer or family recommendations (21, 24, 26). Notably, 53–85% of university students and 60% of community adults in low- and middle-income settings report using antidepressants or sedatives after simply hearing from peers (21, 23). This informal sharing of medicines is particularly dangerous with psychotropics because delayed adverse events (e.g., DIP) may not be immediately attributed to the drug, leading to repeated unsupervised exposure. Strengthening prescription enforcement, pharmacist counseling and public education on the risks of borrowing medications are therefore essential adjuncts to spontaneous reporting systems (20, 26).

Antidepressant-induced pancreatitis most commonly involves agents such as mirtazapine, quetiapine and sertraline (7, 26–30), whereas benzodiazepines (such as clonazepam, previously used by our patient) are rarely implicated. Paroxetine, an SSRI, may precipitate AP through mechanisms involving serotonin-mediated *β*-cell dysfunction, altered gastrointestinal motility and oxidative stress (8, 12–14). In this patient, a combination of direct pancreatic toxicity, immune-mediated hypersensitivity and individual susceptibility may have contributed. Patient education to avoid self-medication and unsupervised drug reuse is essential to prevent recurrence and serious complications.

Recent real-world pharmacovigilance data broaden the DIP spectrum beyond antidepressants. A 2022 FAERS analysis of 1,060 DIP cases confirmed antibiotics, immunosuppressants and antineoplastics as the most frequently reported culprits, while antidepressants remained an uncommon but consistent signal, supporting the idiosyncratic nature of this adverse event (31). Similarly, a 2021 literature review of gliptin-associated pancreatosis underscores the need for class-specific vigilance when drugs target pancreatic exocrine or endocrine pathways (32). Collectively, these findings reinforce that prompt drug withdrawal and patient counseling are the cornerstones of DIP management, even when the implicated agent belongs to a rarely incriminated class.

This study has several limitations. First, comprehensive etiological screening—including MRCP, serum IgG4 subclass, and viral serologies was not performed due to patient refusal after rapid clinical improvement. Therefore, rare causes such as autoimmune or viral pancreatitis cannot be definitively excluded. Second, the positive rechallenge was patient-initiated and not physician-supervised, limiting dose standardization and safety monitoring; as such, it may not fully reflect standard clinical practice. Third, genetic susceptibility factors and pharmacogenomic markers were not assessed, precluding evaluation of individual predisposition to drug-induced pancreatitis. Finally, this report describes a single case from one center, which limits the generalizability of the findings. Further multicenter studies and mechanistic investigations are warranted to clarify causality and elucidate underlying risk factors. Prospective, prescription-registry-linked cohorts are required to quantify the absolute incidence of paroxetine-induced AP and to validate the FAERS signal reported here.

## Conclusion

DIP remains an uncommon but clinically relevant cause of acute AP, often overlooked due to its nonspecific clinical presentation and the predominance of more common etiologies. This case highlights paroxetine as a probable precipitant of AP, evidenced by a strong temporal relationship, symptom recurrence upon patient-initiated rechallenge and rapid resolution following drug withdrawal. Notably, the absence of traditional risk factors and the reproducibility of symptoms upon re-exposure provide compelling support for causality.

Clinicians should maintain a high index of suspicion for DIP in patients presenting with AP of unclear origin, especially when recent medication changes are involved. A systematic diagnostic approach—incorporating thorough medication history, exclusion of common causes and consideration of drug-related timing—is essential for accurate identification. Early recognition and prompt discontinuation of the offending agent are critical for favorable outcomes. The positive outcome in this case further underscores the efficacy of supportive therapy and careful drug management.

Given the widespread use of SSRIs and other antidepressants, awareness of rare but serious adverse events such as DIP is increasingly important. Reporting and documentation of such cases are vital to enhance clinical understanding, inform future pharmacovigilance and guide individualized patient care. Further research is needed to elucidate the underlying mechanisms, identify susceptible populations, develop standardized diagnostic and management strategies for DIP.

In summary, this report adds to the growing body of evidence linking paroxetine to DIP and emphasizes the importance of considering medication-induced causes in patients with unexplained pancreatitis. Vigilance, comprehensive evaluation, and patient education remain key to optimizing outcomes and preventing recurrence.

## Data Availability

The original contributions presented in the study are included in the article/Supplementary material, further inquiries can be directed to the corresponding author.
