# Supplementary material for: Case Report: A case of drug-induced pancreatitis caused by paroxetine with a literature review
Source: Front Med (Lausanne). 2026 Jan 5;12:1688065. doi: 10.3389/fmed.2025.1688065 (PMC12813211; doi:10.3389/fmed.2025.1688065)
Supplement: Supplementary file 1 [file Table_1.DOCX]

**Table S1. Timeline of Clinical Events**

| Date | Days Relative to Admission | Event Description |
| --- | --- | --- |
| March 1, 2024 | -7 | Self-initiated paroxetine 20 mg daily for depression |
| March 2, 2024 | -6 | Developed persistent upper-abdominal pain and bloating (within 24h) |
| March 2–4, 2024 | -6 to -4 | Sought care at local clinic; received symptomatic treatment, IV fluids (transient improvement) |
| March 5, 2024 | -3 | Self-administered paroxetine again (20 mg) |
| March 6, 2024 | -2 | Pain recurred and intensified within 24h |
| March 8, 2024 | 0 | Presented to Xi'an No. 3 Hospital emergency department |
| March 8–11, 2024 | 0 to +3 | Discontinued paroxetine, supportive care started, rapid improvement |
| March 11, 2024 | +3 | Pain resolved, enzymes normalized, repeat CT near-complete resolution |
| March 13, 2024 | +5 | Discharged on trazodone, psychiatric follow-up initiated |
| June 2024 | +90 | Outpatient follow-up: symptom-free, no recurrence, normal labs |

**Table S2. Naranjo Adverse Drug Reaction Probability Scale for This Case**

| **Item** | **Score** | **Rationale** |
| --- | --- | --- |
| Previous conclusive reports | +1 | Multiple published case reports |
| Event appeared after suspected drug administered | +2 | Onset within 24h of paroxetine initiation |
| Reaction improved after drug discontinued | +1 | Rapid resolution after withdrawal |
| Reaction reappeared on readministration | +2 | Recurrence within 24h of self-rechallenge |
| Alternative causes ruled out | 0 | Common causes excluded, rare causes not fully excluded |
| Placebo given | 0 | Not performed |
| Drug detected in blood | 0 | Not performed |
| Severity changed with dose alteration | 0 | Dose unchanged |
| Similar reaction to similar drugs previously | 0 | No previous exposure |
| Adverse event confirmed by objective evidence | +1 | Enzyme elevation, imaging, clear clinical course |
| ****Total**** | ****7**** | ****Probable**** |

**Table S3. WHO-UMC Causality Assessment for This Case**

| **Item** | **Assessment** | **Explanation** |
| --- | --- | --- |
| Time to onset | Compatible | Onset within 24h of drug exposure |
| Dechallenge | Positive | Rapid improvement after withdrawal |
| Rechallenge | Positive | Symptom recurrence upon re-exposure |
| Alternative causes | Unlikely | Common etiologies excluded, rare causes not fully excluded |
| Classification | Probable/Likely | Fulfills most criteria except for complete exclusion of all rare causes |

**Table S4. Common Drugs Implicated in DIP and Their Classification**

| Drug Class | Example Drugs | Class* |
| --- | --- | --- |
| Immunosuppressants | Azathioprine, 6-Mercaptopurine, Tacrolimus | I |
| Diuretics | Furosemide, Hydrochlorothiazide | I |
| Antibiotics | Metronidazole, Tetracyclines, Erythromycin | II |
| Antiepileptics | Valproic acid, Carbamazepine | II |
| SSRIs / SNRIs | Paroxetine, Sertraline, Venlafaxine | III |
| ACE inhibitors / ARBs | Enalapril, Lisinopril, Losartan | III |
| Statins | Atorvastatin, Simvastatin | IV |
| Antipsychotics | Clozapine, Olanzapine | IV |
| Chemotherapeutics | Asparaginase, Cisplatin | I–II |
| Antidiabetics | GLP-1 agonists, Sitagliptin | III |

*Classification Legend:

Class I: Definite association (positive rechallenge, consistent latency, exclusion of other causes)

Class II: Probable association (no rechallenge but consistent latency in multiple cases)

Class III: Possible association (limited reports, less consistent latency or insufficient data)

Class IV: Weak association (inconsistent or conflicting evidence)
